# Reputation-Based Conditional Interaction Supports Cooperation in Well-Mixed Prisoner’s Dilemmas

**DOI:** 10.1371/journal.pone.0036260

**Published:** 2012-05-17

**Authors:** Xiaojie Chen, Alana Schick, Michael Doebeli, Alistair Blachford, Long Wang

**Affiliations:** 1 Evolution and Ecology Program, International Institute for Applied Systems Analysis (IIASA), Laxenburg, Austria; 2 Department of Zoology, University of British Columbia, Vancouver, British Columbia, Canada; 3 Department of Mathematics, University of British Columbia, Vancouver, British Columbia, Canada; 4 Center for Systems and Control, College of Engineering, Peking University, Beijing, China; University of Maribor, Slovenia

## Abstract

In the well-mixed prisoner’s dilemma game, individuals are typically assumed to have no choice about whether to interact with other individuals in the population. In this paper, we instead consider reputation-based conditional interaction and its consequences for the evolution of cooperation. Each individual has a tolerance range, and only interacts with other individuals whose reputation lies within its tolerance range in a chosen sample of the population. Reputation contains information about the number of interaction partners an individual has just cooperated with. We find that the introduction of conditional interaction promotes cooperation in well-mixed populations, and there exist moderate tolerance ranges for which this effect is maximized. For a given tolerance range, there is a critical cost-to-benefit ratio below which cooperation can be promoted. Interestingly, we find that if cooperation evolves, different cooperators’ interaction clusters are typically maintained in the population, each around a different reputation level. We further investigate some properties of these cooperators’ clusters. Moreover, we examine the effects of the sample number on the evolution of cooperation. Our results highlight the importance of the detailed consideration of modes of interaction for the evolution of cooperation in well-mixed populations.

## Introduction

How to understand the emergence of cooperation is a central problem in evolutionary biology [1]–[3]. Evolutionary game theory has become a powerful framework to study this problem [4]. The prisoner’s dilemma game (PDG) is often employed for this purpose [5].

In the PDG framework, a cooperator is an individual who pays a cost *c* that leads to the other individual receiving a benefit *b*, while a defector pays no cost and provides no benefit. In the evolutionary PDG, cost and benefit are measured in terms of payoffs, which are interpreted as reproductive success. In general, the strategy of someone who receives a higher payoff is more likely to be successfully reproduced/propagated [2]. In order for cooperation to increase in a population, cooperative individuals must receive higher payoffs than non-cooperative individuals, which occurs provided cooperators benefit from cooperative acts of others more often than non-cooperative individuals. In other words, there must be positive assortment between cooperative types for cooperation to evolve [5]–[8].

In a well-mixed PDG, all individuals are equally likely to interact with each other (no assortment), defectors have a higher average payoff than unconditional cooperators. Therefore, the relative abundance of defectors increases via natural selection, and drives cooperators to extinction [4], [9]–[11]. In this simple form of PDG, each individual deterministically interacts with all other individuals in the population or with a representative sample of the population. Thus, individuals do not have a choice whether or not to interact with other individuals.

In natural systems, individuals do not always deterministically interact with others in a population. Nor do they interact purely stochastically (as studied in [12]–[15]). A more realistic mode of interaction may be that of selective interaction [16]. Here, we aim to incorporate this selective interaction mode into the well-mixed evolutionary PDG. Individuals’ reputation is a universal and intuitive feature of human society as well as other natural systems, and can be used by other individuals as a selection criterion [17]. Logically, players prefer to interact with others having a high reputation over those with a lower reputation [18], but social tolerance [19] will permit interactions with those having non-high reputation in some cases. They generally have a certain tolerance range, and will myopically interact with those whose reputation is within this range, so that two players can interact only if their reputation levels are both within the other’s tolerance range due to the mutually myopic selections. In this study, we consider the combination of reputation and tolerance range to define the mode of interaction for paired players, and call it conditional interaction.

In previous reputation-based [20]–[24] or tag-based [17], [25]–[27] models, individuals take into consideration the reputations or tags of their opponents to decide whether to cooperate or to defect. The reputation here is used to help individuals choose interaction partners. In fact, information regarding individuals’ reputation can be used as a selection parameter for partner choice [28], further justifying the use of reputation in our model.

In general, an individual’s reputation level is a function of its behavior, increasing with cooperation. As we notice in human society, people are associated with a higher reputation the more they help, or cooperate. In our study, individuals engage in pairwise interactions with others using unconditional strategies, but since the interaction itself is conditional on reputation, different individuals may have different numbers of pairwise interactions. Thus, a cooperator’s number of interaction partners could have a strong influence on its reputation. This is taken into account in the adjustment of individuals’ reputation so that players’ reputation can only be assessed after all the possible interactions are carried out [23].

An individual’s tolerance range is an important parameter, which can help individuals determine their interaction partners. In this study, we mainly examine the effects of tolerance range on the evolution of cooperation in the well-mixed PDG. Intriguingly, we find that moderate tolerance ranges are most beneficial for cooperation in well-mixed populations. Furthermore, for cooperation to evolve, distinct clusters of cooperators are maintained in the population that do not interact with each other.

## Results

**Figure 1:**
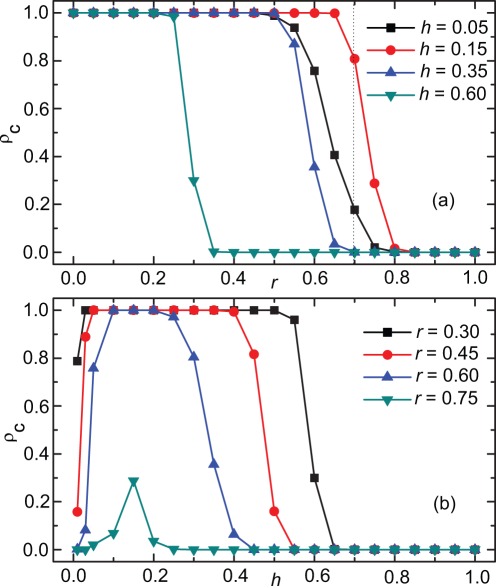
Promotion of cooperation due to reputation-based conditional interaction. Panel (a) shows the cooperation level as a function of *r* for different values of *h*. Panel (b) shows the cooperation level as a function of *h* for different values of *r*. It can be observed that cooperation can evolve even for large value of *r* in the mode of conditional interaction, and there exist intermediate tolerance ranges leading to the optimal cooperation level. Here, *k* is set to 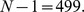

First, we fix the sample size 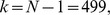 and study the cooperation level as a function of the cost-to-benefit ratio *r* for four different values of the tolerance range *h*, as shown in Fig. 1(a). We see that for each value of *h*, full cooperation is achieved when *r* is small, which is different from previous findings that cooperators easily vanish even for very small *r* in a well-mixed population [4], [11]. This is mainly because that the introduced conditional interaction promotes cooperation, although cooperators still become rapidly extinct as *r* becomes large. Moreover, we notice that, for some fixed *r* [e.g., 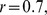 see the dotted line in Fig. 1(a)], the cooperation level for moderate 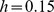 is higher than the cooperation level for other values of *h*, e.g., 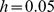 and 0.35. This suggests that there exist some optimal intermediate *h* promoting cooperation.

In order to examine the effect of *h* more precisely, we study the cooperation level 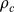 as a function of *h* for various *r* as shown in Fig. 1(b). Interestingly, we find that for the smaller values of *r*, there is a moderate region of *h* resulting in a plateau of full cooperation. When *h* is beyond this region, the cooperation level decreases sharply. The size of this plateau decreases with increasing *r*, and finally vanishes. Even when the plateau of full cooperation is absent, there still exists an optimal *h* leading to the maximal cooperation level [e.g., for 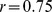 in Fig. 1(b)]. Our results show that such a conditional interaction can provide a positive effect on the promotion of cooperation, which can be restricted by increasing *r*.

**Figure 2:**
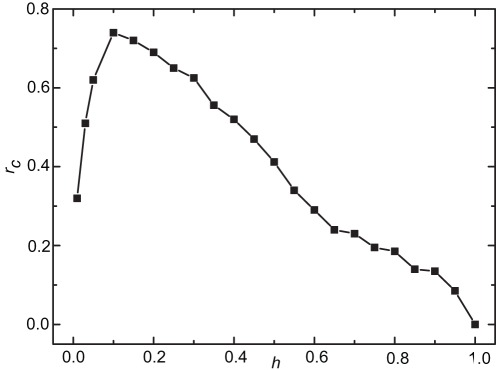
The critical cost-to-benefit ratio 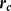 as a function of *h*. Cooperation can be promoted when the cost-to-benefit ratio is less than the corresponding critical value, and 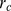 reaches its maximum value at about 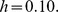 Parameters are the same as those in Fig. 1.

It is worth noting that for each value of *h*, there exists a critical cost-to-benefit ratio 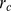 below which cooperation can be promoted. We have determined the critical values 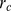 by means of systematic MC simulations, and the results are summarized in Fig. 2. Clearly, we see that 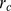 reaches its maximum value at about 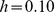, and 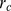 tends to zero if *h* goes to one.

To explain the non-monotonous dependence of 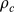 on *h*, we first consider the boundary cases. For 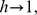 each individual unconditionally interacts with others, and our model recovers the traditional evolutionary PDG model in well-mixed populations. Therefore, defectors can easily take over all the population. For 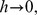 defectors generally have bad reputations and their reputation differences are very slight, hence they do not restrict to interact with each other. Whereas the differences between cooperators’ reputation levels can easily exceed small *h*, so that most cooperators are not permitted to interact with each other. As a result, cooperators do not have the advantage in payoff gained by interacting with each other more often than with defectors, and naturally they are easily wiped out.

**Figure 3:**
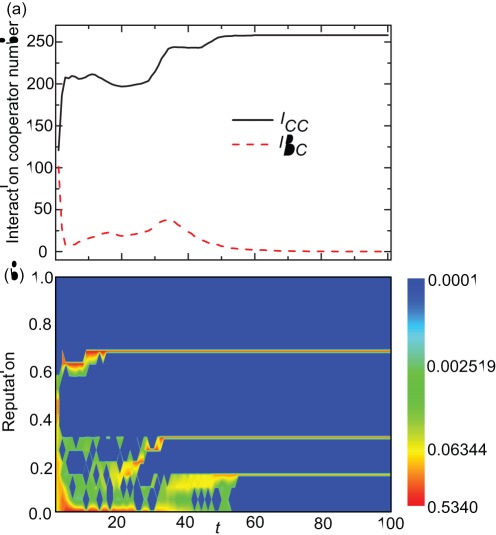
Average interaction cooperator numbers and reputation distribution in the population. Panel (a) depicts the average interaction cooperator numbers of a cooperator (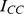) and a defector (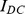) as a function of time step *t*. Panel (b) depicts the contour plot of reputation distribution in the population drawn as a function of time step *t*. It can be observed that during the evolutionary process different cooperators’ clusters are typically maintained in the population, leading to interaction segregation in purely cooperative populations. Here, 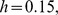 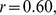 and other parameters are the same as those in Fig. 1.

As *h* departs from zero, the differences between some cooperators’ reputation levels begin to fall within the value of *h*. Thus, cooperators are permitted to interact with some other cooperators [see Fig. 3(a)], resulting in a positive feedback mechanism that enhances the reputation and payoff of cooperators. As time *t* increases, the fraction of individuals having small reputation decreases, and the fraction of individuals having high reputation increases [see Fig. 3(b)]. Interactions among defectors, and interactions between defectors and cooperators are both diminished (see Fig. 3). Importantly, cooperators gradually form stable interaction clusters, where they have very similar reputation levels [see Fig. 3(b)], so that positive assortment between cooperators can be achieved within such clusters, and cooperation can be promoted even in well-mixed populations.

Furthermore, in Fig. 3(b) when the system reaches the absorbing state, i.e., full cooperation, we note that there are still different levels of reputation maintained in the population. This means that even though the population consists of cooperators only, not all individuals, interact, i.e., cooperate, with all other individuals in the population [see Fig. 3(a)]. In other words, the population is segregated into different cooperation clusters, within which cooperation occurs, but between which there is no cooperation. According to the assessment rule, an individual’s reputation not only depends on its strategy, but also depends on the pairwise interaction number. Hence, even if all the individuals choose to cooperate, separate reputation clusters can be maintained in the population. Moreover, we check that reputation clusters can still emerge which promotes the evolution of cooperation for moderate values of tolerance range, in the condition of other initial assignments of reputations.

**Figure 4:**
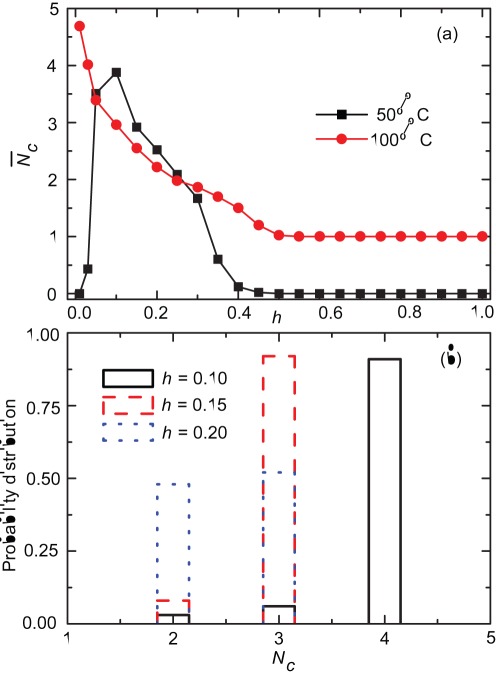
Average number of cooperators’ reputation clusters and their probability distribution. Panel (a) shows the average number of cooperators’ reputation clusters 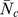 as a function of *h* for two different initial strategy distributions. Panel (b) shows probability distribution of cooperators’ reputation cluster numbers 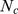 with different values of *h* for initial 50% C. For initial 50% C, the cluster number reaches its maximum value at about 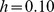 which optimally promotes cooperation. Whereas for initial 100% C, the cluster number decreases with increasing the tolerance range. Here, 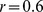 and other parameters are the same as those in Fig. 1.

Some properties of these cooperators’ clusters at equilibrium are shown in Fig. 4. We show the average number of cooperators’ clusters 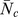 as a function of *h* in Fig. 4(a). For initial 50% C 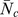 first increases monotonously until reaching the maximum value at about 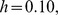 and then decreases with increasing *h*. This hump-shaped dependence can be understood when comparing the number of clusters emerging in populations that are initialized with only cooperators [red dots in Fig. 4(a)]. In that case, 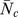 decreases monotonously with increasing *h*, and as is intuitively clear, for small tolerances *h* cooperators would be divided into more and smaller interaction clusters. However, for small *h* defectors can invade and dominate the population (see Fig. 1), which generates the hump shown in the black dots in Fig. 4(a). In Fig. 4(b) we study the probability distribution of 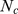 with different intermediate values of *h* for initial 50% C. When full cooperation is achieved there are always at least 2 cooperators’ clusters maintained in the population, and for smaller intermediate *h* larger 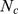 is maintained with a higher probability. Individuals from different cooperative clusters do not interact with each other, but all have the same strategic genotype. These results may reflect the phenomenon of segregation in human society [29]–[32], where individuals who are otherwise genetically similar enough to interact (both cooperators, for example) do not interact with a portion of others in the same community or geographical location based on some types of behavioural tag.

**Figure 5:**
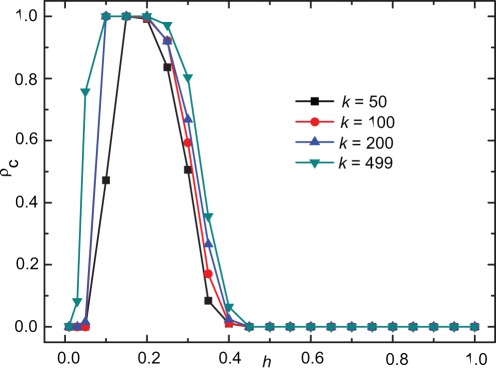
The cooperation level as a function of *h* for different representative sample size *k* of the population. We fix 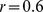 here, and find that the nonmonotonous dependence of cooperation level on the tolerance range does not change qualitatively with the sample number and have checked that the main results can still emerge for other values of *r*.

Finally, we examine the effect of the sample size *k* on the evolution of cooperation. As shown in Fig. 5, we find that for 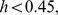 especially for *h* lying in the optimal region, the smaller the sample number is, the lower the cooperation level is. With smaller sample numbers, an additional source of stochasticity is incorporated into individual payoff assignment, which can result in a negative effect on the evolution of cooperation. But the finite population analogue of replicator dynamics we adopted may dismiss some stochastic effects from weak selection [12], [15]. As a result, the nontrivial dependence of cooperation level on the tolerance range does not change qualitatively with the sample number.

## Discussion

Reputation is used as a phenotypic feature for selective interactions in our model, and such reputation-based selective interactions differ from previous explicit ones [2], [20]–[24]. In general, indirect reciprocity works through reputation. Individuals choose to cooperate or to defect, depending on the recipient’s reputation and their own, and those with high reputation are more likely to receive help from others. In our model, it is only when individuals determine whether to participate that they consider the opponent’s reputation and ones own, and they play the same strategy with all interaction partners. Individuals’ reputation generates phenotypic assortment for preferential interactions. In particular, cooperators maintain a non-zero reputation level, while the reputation of defectors decreases to zero. This generates the positive assortment between cooperators necessary for the maintenance of cooperation.

Our assessment rule gives individuals a reputation that varies from zero to one, which differentiates the players in the well-mixed populations. It is interesting to note that even if cooperation dominates, not all cooperative individuals have the same reputation level. Rather, the cooperators form interaction clusters based on different reputation levels, with cooperative interactions taking place within clusters, but not between different clusters. Such cluster formation within populations consisting entirely of cooperators does not seem to have been observed in previous reputation-based studies [2], . But interestingly, such similar finding has been experimentally demonstrated in other relevant works regarding in-group favoritism [29]–[32], where cooperators only help others in their group. In this sense, our work complements and confirms previous results by introducing such a reputation-based conditional interaction rule. Moreover, it is worth pointing out this assessment rule of reputation is set by only taking into account some limited factors. In fact, based on this assessment rule, some more factors (e.g., an individual’s reputation should keep decreasing after playing as a defector in some interactions) can be incorporated into the evaluation of reputation, which could be further helpful in studying the evolution of cooperation in more realistic systems.

Because the reputation of individuals in our model can be seen as a kind of tag, our model is related to the considerable body of work on tag-based cooperation, according to which cooperators prefer to help others with similar tags [17], [25]–[27], [33]–[37]. In general, those tag-based selective interactions are based on fixed tags, and often a result of self-similarity mechanism in nature. Individuals (or altruists) play a conditional strategy in the framework of compulsory participation: they cooperate with all individuals who are close enough in tag space and defect otherwise. This selective interaction between similar players is active. Whereas the conditional interaction rule in our model results from individuals’ respective preferences, so that individuals passively interact with others having similar and dynamical reputation level by using pure strategies. In addition, the phenotypic (heritable) signals in tag-based models are possibly less reliable [27], [33]. Defectors may detect this tag, and destroy the tag-based cooperation. Presently, individuals’ reputation is adjustable based on past actions, which could facilitate preferential assortment. In particular, cooperators’ reputation can become very similar with some others’ at moderate tolerance range. The positive correlation between reputation and moderate tolerance range leads to positive assortment between cooperative types–the basic mechanism that promotes cooperation [5]–[8]. Noticeably, we also show that in contrast to previous related works [34], [36], this promotion does not need the additional requirement of spatial population distribution.

The parameter *h* in our models could be used to characterize the strength of individuals’ rationality at choosing interaction partners. For 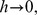 individuals only tend to interact with those having higher reputation levels, demonstrating a rational performance; whereas for 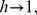 individuals unconditionally interact with other individuals, performing randomly. It is important to emphasize that such kind of rationality is mainly based on individuals’ reputation information, rather than payoff information. Indeed, the bounded rationality of individuals has been taken into account in the dynamics of games in real systems, and may have different forms [17]. For example, in [38]–[43], a stochastic Fermi function evolutionary rule is presented to capture the bounded rationality of individuals. Here we capture the bounded rationality of individuals in another sophisticated situation, and we find that this different form of bounded rationality is beneficial for the altruistic behavior, consistent with previous results [39], [40]. In this sense, our work further shows that individuals’ bounded rationality can become a beneficial characteristic in understanding cooperative behavior in the real world [17].

We observe that individuals are constrained to interact with few others, and cooperators’ interaction clusters can be formed. These observations are conceptually analogous to the ones in games on graphs, where individuals are constrained to interact with few others along the edges [44]–[46]. Furthermore, there are some cooperators with high reputation during the evolutionary process, who act as hub cooperators in heterogeneous networks [47]–[51]: they can help others more, resist the exploitation by defectors, and breed other potential cooperators in the population. By means of this analogy, we further confirm that an optimal environment for the evolution of cooperation is warranted by the middle range of tolerance.

It is noteworthy that our approach bears some similarity with game models about the coevolution of strategy and network structure (see [52], [53] for review papers), albeit presently only the interaction network is dynamical. Individuals can have different and dynamical pairwise interaction numbers by this mode of selective interaction. In particular, individuals will refuse to participate in the interactions if they are dissatisfied with their parters. This similar feature has been reflected in some previous works about evolutionary games in dynamical networks. For example, in [54] if a player is dissatisfied with the interaction, then it competes with the partner to rewire the link; and in [55] undesired links (the links between cooperators and defectors) break fasters under the proposed active linking rule. Under this approach, dynamical heterogeneous interactions emerge in the well-mixed populations, and allow cooperation to spread at moderate tolerance range. Moreover, some individuals may be isolated by all others, and become interaction loners. However, the loner here is not a strategy choice and does not arise voluntarily [56], [57].

In summary, we have presented a conditional interaction rule in the well-mixed prisoner’s dilemma game, which combines individuals’ social tolerance range with reputation information to help individuals choose interaction partners. We have mainly studied the effects of tolerance range on the evolution of cooperation. Interestingly, we found that there exist optimal moderate tolerance ranges leading to the highest levels of cooperation, and the conditional interaction rule can be helpful in solving the problem of cooperation in well-mixed populations. Furthermore, we obtained the critical cost-to-benefit ratio below which cooperation can be promoted for each value of tolerance range. Also, we found that when cooperation evolves, different cooperators’ clusters are typically maintained in the population, leading to interaction segregation in purely cooperative populations. When full cooperation is achieved, there are always at least two cooperators’ clusters presenting in the population, and the number of different cooperation clusters appears to be maximal near the tolerance range that optimally promotes cooperation. Thus, our model not only shows that conditional interaction is an alternative way to solve the problem of cooperation through assortment, but also suggests a mechanism for generating interaction segregation within cooperative populations. In future work, it would be interesting to consider these mechanisms in other evolutionary games, e.g. in the snowdrift game [5], and to consider the evolutionary dynamics of tolerance levels.

## Materials and Methods

Consider the evolutionary PDG in a well-mixed population with size *N*. Each individual *x* can either cooperate (C) or defect (D). The strategy of player *x* is denoted by 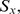 where 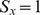 corresponds to the strategy C, and 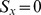 to the strategy D. Following previous works [44], [58], we adopt the rescaled payoff matrix depending on one single parameter so that the problem can be simplified
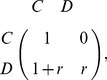
(1)where 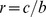 represents the cost-to-benefit ratio. It is worth pointing out that the qualitative results do not change if we adopt other forms of payoff matrix for PDG [48], [59].

In well-mixed populations, each player chooses a representative sample of the population as the possible interaction partners in each generation [15]. Without loss of generality, we assume that there are *k* (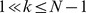) individuals in the chosen subset of the population. Note that if 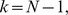 each individual has the opportunity to interact with all others in the population at each generation.

Then, player *x* will compare its current reputation with player *y*’s in the chosen sample of the population. It will be willing to interact with player *y*, if player *y*’s reputation is within its tolerance range, that is,
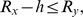
where 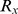 (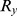) is the current reputation level of player *x* (*y*), and *h* is the tolerance range of player *x*. In this study, we assume that each player has the same tolerance range. Similarly, player *y* will be willing to interact with player *x* if 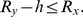 As a result, only when 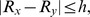 player *x* can interact with player *y*, and obtain the payoff from the game.

According to the above interaction rule, player *x* chooses its interaction partners from the sample of the population, and collects its payoff 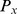 by using strategy 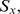


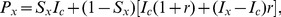
(2)where 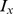 is the number of player *x*’s interaction partners, 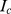 is the number of cooperators among the interaction partners, and 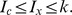


Following the interactions with the sample of the population, player *x*’s reputation level is adjusted, and its updated reputation level, 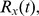 is a weighted combination of its previous level at time 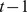 and its actions taken against interaction partners at *t*. Formally,
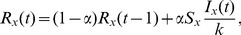
(3)where 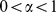 is a weighting factor. For 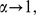 individuals’ reputation depends only on the present interactions. For 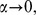 individuals’ reputation is unchanged. Without loss of generality, the second term in Eq. (3) is normalized (divided by the number of the sample *k*), and is a measure of how much player *x* cooperated with others. In this study, the reputation level for *N* agents is initially random and can vary between 0 and 1, and correspondingly the tolerance rang is set to 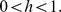


Following the reassignment of reputation levels, player *x* selects another player *y* randomly from the entire population for strategy updating. Whenever 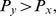 it will adopt the selected individual *y*’s strategy with probability given by
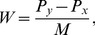
(4)where *M* ensures the proper normalization and is given by the maximum possible difference between the payoffs of *x* and *y*. It is important to point out that the selection under such strategy update rule is strong and can dismiss some stochastic effects, so that we can focus more forcefully on the effects of the reputation-based conditional interactions.

In this study, we set 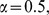 and investigate the effects of *h* on the evolution of cooperation. Moreover, we have checked that the main results remain unaffected qualitatively when changing α within realistic limits.

We study this model by Monte Carlo (MC) simulations, which are carried out in the well-mixed population with size 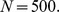 Initially, the two strategies of C and D are randomly distributed among the population with an equal probability, and individuals’ reputation levels are randomly distributed within the interval 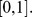 Under stochastic dynamics, the population will inevitably converge to one of the two possible absorbing states: 100% cooperators or 100% defectors [54], [60]. In our study, we run 500 independent simulations for each set of parameters, and compute the fraction of times that the system evolves to 100% cooperators as the cooperation level 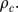 However, we find that the time for reaching an absorbing state may be prohibitively long for certain sets of parameters. If the population does not converge to an absorbing state after 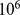 generations, the cooperation level is determined by the average fraction of cooperators in the population over the last 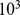 generations. Furthermore, we implement this computational model with synchronous updates [46].
